# Errata

**DOI:** 10.36660/abc.20201048

**Published:** 2020-10-13

**Edição de Julho de 2020, vol. 115 (1), págs. 102-108**

No Artigo Original “Quercetina Melhora o Perfil Lipídico e Apolipoproteico em Ratos Tratados com Glicocorticóides em Altas Doses” com, número de DOI: https://doi.org/10.36660/abc.20180397, publicado no periódico Arquivos Brasileiros de Cardiologia, 115(1):102-108, na página 102, incluir mais uma afiliação para o autor Ahmad Reza Dehpour. Incluir a instituição: Experimental Medicine Research Center, Tehran University of Medical Sciences, Tehran, Irã.

**Edição de Agosto de 2020, vol. 115 (2), págs. 229-237**

No Artigo Original “Revascularização Completa Versus Tratamento da Artéria Culpada no Infarto com Supradesnivelamento do Segmento ST: Registro Multicêntrico” com, número de DOI: https://doi.org/10.36660/abc.20180346, publicado no periódico Arquivos Brasileiros de Cardiologia, 115(2):229-237, na página 229, corrigir o nome do autor Alexandre Tognon para: Alexandre Pereira Tognon e autor Rogério Tumelero para: Rogério Tadeu Tumelero. Retirar a instituição: Universidade de Passo Fundo, Passo Fundo, RS - Brasil e alterar para: Associação Hospitalar Beneficente São Vicente de Paulo, Passo Fundo, RS - Brasil das afiliações dos autores Alexandre Pereira Tognon e Rogério Tadeu Tumelero.

