# Supplementary material for: Errata
Source: Arq Bras Cardiol. 2020 Oct 13;115(4):800. [Article in Portuguese] doi: 10.36660/abc.20201048 (PMC8386970; doi:10.36660/abc.20201048)
Supplement: Translation

# Translation: Erratum

**July 2020 Issue, vol. 115 (1), pages 102-108**

In the Original Article “Quercetin Ameliorates Lipid and Apolipoprotein Profile in High-Dose Glucocorticoid Treated Rats”, with DOI number: https://doi.org/10.36660/abc.20180397, published in the periodical Arquivos Brasileiros de Cardiologia, 115(1):102-108, on page 102, add one more affiliation for the author Ahmad Reza Dehpour. Include the institution: Experimental Medicine Research Center, Tehran University of Medical Sciences, Tehran, Iran.

**August 2020 Issue, vol. 115 (2), pages 229-237**

In the Original Article “Complete Revascularization Versus Treatment of the Culprit Artery Only in ST Elevation Myocardial Infarction: A Multicenter Registry”, with DOI: https://doi.org/10.36660/abc.20180346, published in the periodical Arquivos Brasileiros de Cardiologia, 115(2):229-237, on page 229, correct author name Alexandre Tognon to: Alexandre Pereira Tognon and the author Rogério Tumelero to: Rogério Tadeu Tumelero. Remove the institution: Universidade de Passo Fundo, Passo Fundo, RS - Brazil and change to: Associação Hospitalar Beneficente São Vicente de Paulo, Passo Fundo, RS - Brazil from the affiliations of the authors Alexandre Pereira Tognon and Rogério Tadeu Tumelero.
